# Exploring the biodiversity of cetacean communities along the western North Atlantic Ocean shelf-break

**DOI:** 10.1098/rsos.241658

**Published:** 2025-07-02

**Authors:** Samara Haver, Peter Corkeron, Annamaria DeAngelis, Simone Baumann-Pickering, Danielle Cholewiak, Genevieve Davis, Kaitlin Frasier, Natalie Posdaljian, Macey Kadifa, Alba Solsona-Berga, Annabel Westell, Sofie Van Parijs

**Affiliations:** ^1^ Cooperative Institute for Marine Ecosystem and Resources Studies, Oregon State University, Newport, OR, USA; ^2^ NOAA Pacific Marine Environmental Laboratory, Newport, OR, USA; ^3^ Griffith University, Brisbane, Queensland, Australia; ^4^ NOAA Fisheries Northeast Fisheries Science Center, Woods Hole, MA, USA; ^5^ Scripps Institution of Oceanography, University of California San Diego, La Jolla, CA, USA; ^6^ Integrated Statistics Inc., under contract to the Northeast Fisheries Science Center, NOAA Fisheries, Woods Hole, MA, USA

**Keywords:** passive acoustic monitoring, cetaceans, long-term monitoring, North Atlantic ocean, biodiversity

## Abstract

Declining biodiversity is a global issue that encompasses reduced species abundance and changing distributions. Observing community groups can reveal spatial patterns and identify shifts in presence over time, providing information to support conservation of biodiversity and ecosystem services. Passive acoustic monitoring (PAM) is a tool for observing ocean environments, and coupled with species-specific detectors and classifiers can provide information about cetacean communities. This study analysed data collected by 10 PAM recorders deployed along the western North Atlantic shelf break between April 2016 and June 2019. Relative acoustic presence of 13 specific cetacean species and a grouped category for delphinid species was evaluated using biodiversity metrics to compare dissimilarity of community composition across the sampling locations. In all years, presence of Gervais’ beaked whale (*Mesoplodon europaeus*) was the primary distinguishing factor in grouping sites, while detections of other beaked whale species were of secondary importance, followed by other odontocetes. The presence of mysticetes also varied by site and season, and co-occurrence comparisons revealed distinctive communities at each site. This study highlights the significance of identifying beaked whales to species rather than grouping them, providing insights into the dynamics of cetacean populations to inform management and conservation of these important species.

## 1. Introduction

Healthy biomes, both terrestrial and marine, consist of communities of living organisms that have common characteristics adapted to the ecosystem that they inhabit. Loss of species biodiversity in many regions of the world is harmful to the function and resilience of marine, terrestrial and freshwater ecosystems and is increasingly being recognized as a critical global issue [1,2]. Biodiversity loss can be observed in the increase of endangered or protected species concurrent with decreasing population sizes of foundation species. Together, these changes indicate an ecological crisis wherein extinction rates are anticipated to severely impact global environments, compromising ecosystem services to nature and society [3]. For decades, the United Nations has implemented international agreements related to conserving global biological diversity that include steps to protect and restore natural systems to mitigate species extinction [4]. Many of these treaties emphasize ocean conservation, including a historic agreement in 2023 to conserve biodiversity in international waters [5].

In order to make data-driven decisions to conserve species and promote biodiversity, policy makers and managers need reliable data and decision-making tools to track local and global impacts of changes in species and environments. For example, leading up to the UN agreement on marine biodiversity, multiple countries developed concurrent-focused policy to conserve marine species and maintain biodiversity in response to specific threats such as ocean noise. For example, the European Union Marine Strategy Framework Directive [6] was adopted with goals to develop noise monitoring programs and limit the adverse effects of anthropogenic noise in marine habitats [7]. Similarly, the United States and Canada put forth respective Ocean Noise Strategies which support resource monitoring and conservation [8,9]. These policies share prioritization of conserving cetacean species and populations, informed by research that has demonstrated the negative impacts of ocean noise on cetacean life history. In concert with concurrent efforts to mitigate other threats, regulators and scientists are working towards meeting the goals of these policies to support healthy marine ecosystems.

In 2022, the United States (U.S.) Executive Office of Science and Technology Policy outlined the first U.S. National Nature Assessment, directing federal agencies to evaluate conditions of US natural resources and identify opportunities for conservation [10]. Assessing biodiversity in marine ecosystems is specifically of high interest to the U.S. National Oceanic and Atmospheric Administration (NOAA) and the U.S. Navy because of the overlap of major fisheries, commercial activity and national security with protected species and marine habitats, and shared priorities between agencies include endangered species conservation and monitoring. Researchers and policy makers also seek new information to inform species protections, such as for the many species of beaked whales (*Ziphiidae*), most of which have unknown population trends and are considered to be highly sensitive to anthropogenic sounds. Species monitoring efforts to assess biodiversity are highly prioritized in federally protected habitats, such as U.S. National Marine Sanctuaries and Monuments, where monitoring can provide important information for managers about ecosystem health.

Marine shelf-break environments mark the point at which the seafloor transitions between the relatively shallow continental shelf and the continental slope. They can be highly productive areas of upwelling, governed by seasonal variations in biological and physical processes, and form important habitats for many marine species. Collecting long-term monitoring data to assess biodiversity in these offshore locations is challenging, especially on the East Coast of the United States. Thus, marine ecosystem monitoring is accomplished through a myriad of tools and means. Passive acoustic monitoring (PAM) provides a non-invasive method for long-term sampling of sound conditions and species occurrence in underwater environments.

Autonomous PAM recorders can be used to collect information about long-term trends of ocean sound and the presence of soniferous species without the high cost of vessel surveys, continuous disturbance or limitations of weather and daylight [11]. Analysis of PAM data sampled in terrestrial environments has been shown to be an accurate and valuable method to determine species presence without some of the disadvantages of traditional survey methods, such as observer bias or the short-term nature of human-staffed field visits [12,13]. As many marine animals rely on sound for essential life functions, the efficiency of PAM can be utilized to monitor spatial and temporal acoustic presence of different species [14,15]. Many cetacean species are known to rely on acoustic communication, and PAM is established as a reliable method for determining the presence of highly vocal whale and dolphin species on fine scales [16]. Even more cryptic marine mammal species (e.g. beaked whales) rely on sound for essential life functions and can be observed via PAM [17–19].

The abilities and extent of underwater PAM recordings continue to grow as capacity of recording technologies advance, and researchers are now able to utilize continuous PAM data to monitor individual and groups of species over a broad range of frequencies for applications such as tracking seasonal presence in a specific region [20] or following migratory movement over thousands of kilometres [21]. However, although PAM continues to provide rich datasets for resource monitoring, many tools available to quantify biodiversity have not yet been applied to PAM data. In part, constraints of time-intensive analysis of continuous long-term datasets have made it difficult to apply more complex and data-hungry measures of biodiversity, such as dissimilarity and co-occurrence [22]. However, extracting species-specific results from PAM data provides a valuable resource to monitor soundscapes and measure ecological resilience of species and groups of species in environments [23], and thus is valuable to develop tools to simplify and expedite these analyses.

Monitoring groups of species and the diversity within them is an important aspect of biodiversity. The effective number of species (ENS) quantifies both the richness or number of species present and the evenness or proportional presence of those species, and can be measured from PAM detection data [24]. By considering not just the number of species present, but also their relative presence, the ENS is an efficient method to monitor populations and evaluate ecosystem function spatially and at different times. For community assemblage analysis, the species exchange ratio (SER) is a biodiversity metric that targets changes in species composition over time. Richness- and abundance-based SER measurements (SERr and SERa, respectively) quantify the turnover of species by considering the ratio of species gained and lost within a specific timeframe. Combined, the SERr and SERa indices provide information on the turnover of species within ecosystems. Here, we apply these metrics to PAM data alongside conditional inference trees and acoustic niche visualizations to demonstrate the applications of PAM data to describe community composition and support biodiversity assessments. These results can be further paired with other data streams, such as visual surveys, to increase the accuracy and breadth of cetacean monitoring efforts.

In this article, we analyse a 3-year dataset (2016–2019) of continuous PAM data sampled across a broad frequency range (10 Hz–100 kHz) at 10 sites along the western North Atlantic shelf break for the acoustic presence of cetacean species. We then apply established biodiversity metrics to the species presence results to evaluate temporal and spatial differences in community assemblages across the sites. By applying these tools to PAM data, we can identify the composition of multi-species cetacean groups as well as the species that define the community composition clusters that differentiate the sites. Our evaluation of community-specific questions aims to reveal how habitats host different assemblages of species and identify important areas for multi-species communities. Using PAM tools allows us to collect data over extended durations which can be used to provide information about large-scale species community composition. Pairing these methods provides complementary comparisons of species communities, each providing important information to enrich our overall understanding of animal presence across space and time.

## 2. Methods

### 2.1. Acoustic data collection

**Figure 1.**
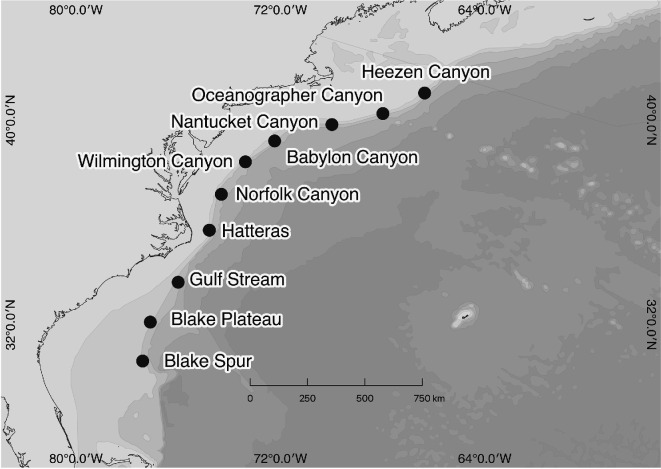
Map of the deployment locations of the 10 HARP on the shelf break of the western North Atlantic Ocean. From north to south the sites are: Heezen Canyon, Oceanographer Canyon, Nantucket Canyon, Babylon Canyon, Wilmington Canyon, Norfolk Canyon, Hatteras, Gulf Stream, Blake Plateau and Blake Spur.

Passive acoustic data were sampled by an array of 10 high-frequency autonomous recording packages (HARP) deployed at 10 sites spaced 130−230 km apart between 30°0′N and 42°0′N along the shelf-break in the western North Atlantic Ocean (figure 1). Each HARP consisted of two calibrated omni-directional hydrophones and a 16 bit data acquisition system to record continuously. One hydrophone recorded at a sampling rate of 200 kHz with a 10 Hz–100 kHz frequency response, and a second lower frequency hydrophone sampled at 2 kHz with a 10 Hz–1 kHz frequency response (for further specifics, see [17]). Each HARP was deployed for up to 14.5 months, and was recovered and redeployed up to three times between 2016 and 2019 for continuous monitoring [17]. The deployment depth of each HARP varied between 450 and 1350 m depending on the latitude of the recorder (for detailed locations and depths, see table 1 in [17] ).

### 2.2. Species detection and classification

A combination of species-specific detectors were used to identify the daily presence of 13 cetacean species (five mysticete species and eight odontocete species) in the PAM data. The five species of mysticetes included in analyses were: blue (*Balaenoptera musculus*), fin (*Balaenoptera physalus*), humpback (*Megaptera novaeangliae*), North Atlantic right (*Eubalaena glacialis*) and sei whales (*Balaenoptera borealis*) (see [25] for data). All mysticete species vocalizations were detected and classified using an automated detector, the low-frequency detection and classification system, which identifies individual vocalizations by drawing pitch tracks through high-energy tonal sounds and utilizes multivariate discrimination to classify them using a customized call library [14,26]. Automated detections of vocalizations for each species were manually verified and summarized into daily time bins by species for further analysis, following protocols from Davis *et al*. [27] and Davis *et al*. [14].

Detection and classification of odontocetes included five beaked whale species: Blainville’s beaked whale (*Mesoplodon densirostris*), goose-beaked whale (*Ziphius cavirostris*), Gervais’ beaked whale (*Mesoplodon europaeus*), True’s beaked whale (*Mesoplodon mirus*) and Sowerby’s beaked whale (*Mesoplodon bidens*). At times, it is difficult to distinguish between Gervais’ and True’s beaked whale clicks due to spectral similarities, thus a mixed category of both species was also included. A machine learning pipeline, starting with the SPICE-Detector Remora (https://github.com/MarineBioAcousticsRC/Triton/wiki/SPICE-Detector) within the acoustic analysis program Triton [28] was applied to all datasets to detect beaked whale clicks. A neural net classifier (29) with 0.99 confidence threshold was then applied to the processed data (except Norfolk Canyon, which was manually analysed as a training dataset). Species-specific detections were manually validated to daily level with DetEdit in Matlab 2016b [30] and summarized detections of >10 validated clicks within a minute were grouped to confirm daily presence [31].

The Triton SPICE-Detector Remora was also applied to all datasets to detect daily acoustic presence of Kogia species, Risso’s dolphin (*Grampus griseus*) and grouped delphinid species [32]. All detections were manually reviewed in Triton to confirm daily presence. Sperm whale (*Physeter macrocephalus*) echolocation clicks were detected via an automated multi-step algorithm within Matlab, and detections were verified based on received energy level and frequency characteristics [33]. Sperm whale detections were summarized in daily bins for comparative analysis.

### 2.3. Biodiversity analysis

#### 2.3.1. Relative acoustic presence

Binary daily acoustic detections for each of the 13 cetacean species and grouped unknown delphinid species included in this analysis were summarized into a monthly relative acoustic presence value using the days of occurrence to obtain a relative presence measurement, that is, a species recorded every day in a month has a higher relative presence value compared to a species detected only once in a month. Due to differences in bioacoustic activity across the included species, monthly time bins are preferred over daily time bins because the presence or absence of a species on any given day can be stochastic, artificially inflating turnover estimates. All available data for each site was included in the analysis.

#### 2.3.2. Analysis of turnover

Species turnover at each site over time was quantified in R software [34] using ENS and richness indices following Hillebrand *et al*. [24] and Van Opzeeland & Hillebrand [22]. Specifically, the variability of communities at each site was quantified by measuring two similar SERs. The SERr is congruent with the Jaccard dissimilarity index and thus based on presence–absence only and measures monthly exchange in species identity (i.e. taxonomic classification). Specifically, the SERr is quantified by comparing the number of newly detected species (i.e. immigrating species) and number of species no longer detected in comparison to the previous month as a fraction of the total number of species in both months [24].

The SERa is a measure of turnover that also takes into account changes in species presence based on the proportion of days in a month a species was detected. SERa is congruent with the Wishart dissimilarity metric and weights dominance as the Simpson-based ENS ([24]; see appendix S3). Variation of SERa was used to assess a seasonal shift in ‘dominance’; that is, if some species have a more significant acoustic presence during some months of the year and then are either less acoustically active or leave the area. Both SERr and SERa approach 0 if the identity of a species and the dominance structure do not change, and approach 1 if all species are replaced or shifted in their dominance. SERa will reduce to SERr when there is no difference in the relative abundance of each species.

#### 2.3.3. Conditional inference trees

Conditional inference trees (Ctrees [35]) were modelled from days present per month for each of the 13 individual species and grouped delphinid species at each of the 10 study sites. The Ctrees identify specific species that may be driving dissimilarity in community groups over time and space as individual nodes within the tree. The values on the lines joining nodes indicate the monthly presence of the species named in the node in each direction of the split. Pie plots at the terminal nodes of the Ctree show the probabilities of one or a combination of sites which host the species names in the branch nodes. Thus, the end nodes visualize similarities between sites based on the occurrence and grouping of certain species. Other (not-named) species may also co-occur at the sites, but the species named in the node are identified by the Ctree model as more significant for distinguishing the sites.

The Ctrees were generated using the R packages party [35] and party kit [36], and the R package ggparty [37] was used to plot the trees. Three Ctree models were generated from the dataset, one including all detections of all species without depth limitations (full dataset), one including all detections of all species that was limited to a single node (stump), and a third that excluded all detections of odontocete species to visualize the presence of highly migratory mysticete species across the 10 sites.

#### 2.3.4. Acoustic niche visualization

An acoustic niche visualization was generated for all available species detections over the 3-year dataset. The visualization was based on the spectrographic box display plots introduced by Van Opzeeland & Boebel [38] and customized for species present in the North Atlantic (Georges Bank) by Weiss *et al*. [39]. Daily presence of each cetacean species is colour-coded according to the species and shaded only in the frequency band that the animal is known to call (for a detailed table, see [39]). The acoustic niche plot visualizes the potential frequency overlap of species’ vocalizations that were detected on the same day.

## 3. Results

### 3.1. Relative acoustic presence

**Figure 2.**
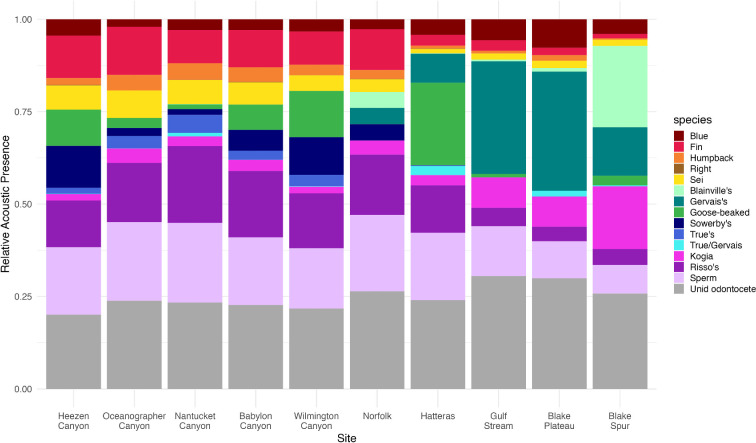
Relative acoustic presence of 13 known cetacean species and grouped unknown odontocetes (delphinids) at 10 North Atlantic recording sites named from north (left) to south (right). Relative acoustic presence was normalized to a scale of 0–100% by averaging the number of days per month each species was detected over 3 years of data collection. Mysticete species are labeled with red-orange colours, beaked whale species are shades of green and other odontocetes are labelled with blue-purple colours.

Relative acoustic presence of the 13 species and grouped delphinid species varied across the 10 study sites (figure 2). Detections of mysticete species were similar from the northernmost site, Heezen Canyon, down to Norfolk, where they made up approximately 25% of the total bioacoustic detections summarized from 3 years of acoustic data. At those sites, fin whales were the most prevalent, followed by sei, humpback and blue whales (respectively). Mysticete species were comparatively less prevalent (~10–15%) at the four sites south of Norfolk (Hatteras, Gulf Stream, Blake Plateau and Blake Spur). At those sites, blue whales were most prevalent, followed by fin and sei whales. Humpback whales were comparatively less prevalent than at the six sites to the north. North Atlantic right whales were rarely detected at any of the included sites.

Relative acoustic presence of beaked whale species varied most across sites. Results suggest each site had a unique community of species and prevalence of those species, with a maximum of four of the five possible beaked whale species detected at each site. At the five northernmost sites, primarily Sowerby’s, True’s and goose-beaked whales were detected, as well as a mixed category of either True’s or Gervais' beaked whales. On one day at Babylon Canyon, Gervais’ beaked whales were detected separately from the True’s or Gervais' mixed category. These three beaked whale species were most prevalent (25%) at Heezen and Wilmington Canyons, slightly less prevalent at Babylon Canyon (~20%), and least prevalent at Oceanographer and Nantucket Canyons (<10%). Among those sites, Sowerby’s and goose-beaked whales were approximately equally acoustically abundant, with a smaller percentage of True’s beaked whales detected at Heezen, Babylon and Wilmington Canyons. At Oceanographer Canyon, acoustic detections of Sowerby’s, True’s, and goose-beaked whale clicks were approximately equal. Overall, True’s beaked whales were more prevalent than all other beaked whale species at Nantucket Canyon compared to the other nine sites.

Blainville’s and Gervais' beaked whales were only detected at Norfolk Canyon and the four sites to the south. Other species detected at those five sites were Sowerby’s (Norfolk), goose-beaked (Hatteras and Blake Spur) and a mixed category of either True’s or Gervais' beaked whales (Hatteras, Blake Plateau and Blake Spur). Relative acoustic presence among beaked whale species was most even at Norfolk Canyon with approximately equal presence of Blainville’s, Gervais' and Sowerby’s beaked whales.

The family of beaked whales were most prevalent at sites south of Norfolk (Hatteras, Gulf Stream, Blake Plateau and Blake Spur). At Hatteras, goose-beaked whales were more present (~20%) than other beaked whale species (~10%). Gervais' beaked whales were most present at the Gulf Stream and Blake Plateau sites, where detections showed ~30% relative acoustic presence, more than any other individual species. Gervais' beaked whales were also present at Blake Spur (~15%), but not more than Blainville’s beaked whales which were approximately 25% of relative acoustic presence.

Relative acoustic presence of Kogia species and Risso’s dolphin were similar for the seven sites from Heezen Canyon to Hatteras, with a higher proportion of Risso’s dolphin than Kogia species (figure 2). At the three southernmost sites, Gulf Stream, Blake Plateau and Blake Spur, Kogia species were more present than Risso’s dolphin. At those three sites, sperm whales were also slightly less abundant (~10–15%) compared to the seven sites to the north (~15–20%). Across all sites, grouped unknown odontocetes (delphinids) were similarly abundant (~20–25%).

### 3.2. Analysis of turnover

**Figure 3.**
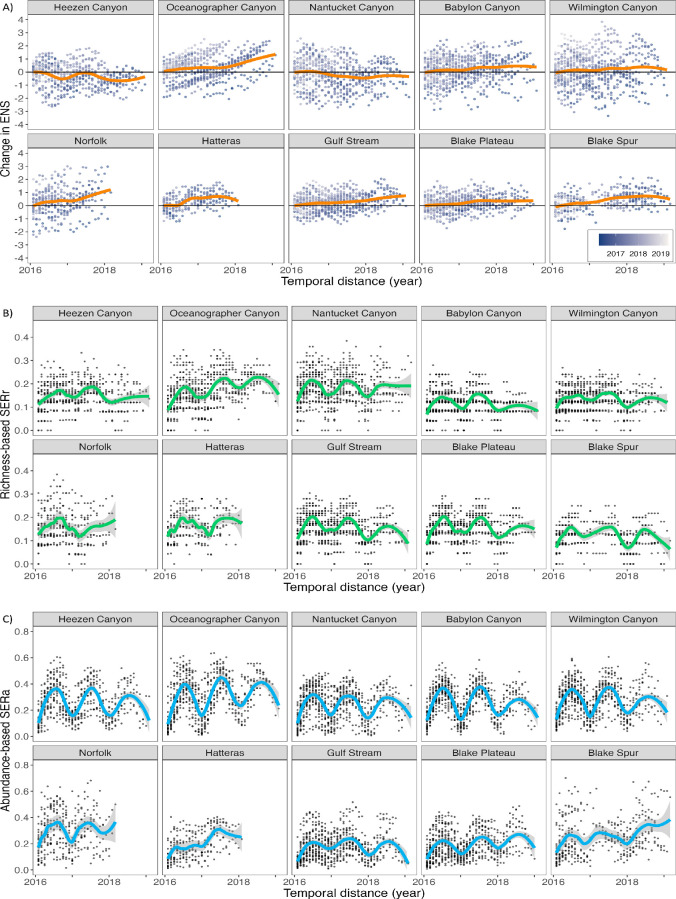
Site-specific community composition trends for 10 North Atlantic monitoring sites with increasing temporal distance over 3 years of acoustic data collection. In all plots, data points represent the change in number of days per month that a single species was detected compared to the previous month for each species. A bold solid coloured line (LOESS function, span 0.25) is overlaid on data points to illustrate patterns. (A) Monthly effective number of equally abundant species (ENS) detected between 2016 and 2019 at 10 monitoring sites in the North Atlantic. (B) Monthly identity turnover of SERr. (C) Monthly turnover of SERa based on the proportion of days in a month a species was detected.

The monthly change of days per month that we detected each species at each site was relatively consistent throughout the 3-year dataset, with an average month-to-month change in the ENS of 0−2 at all sites (figure 3A). Overall, the change in ENS has a slightly larger range at the more northern sites, and although the location of some of the sites moved slightly between deployment years, they were all maintained at an approximate slope location. The most positive shift in ENS across all sites was at Oceanographer Canyon, where a slight increase was observed over time. Only Heezen and Nantucket Canyons displayed a negative trend of change in ENS, with the largest change being −1 ENS. At the other seven sites, change in ENS was balanced between positive and negative month-over-month changes which resulted in a mostly flat trendline that averaged an ENS change of ~0–1 over the 3-year study duration.

The SERs for richness and abundance provided additional information about the temporal trends of the community assemblages at each site (figure 3B,C). At some sites (e.g. Nantucket Canyon) there are more pronounced seasonal turnover trends, while at others (e.g. Blake Spur) we observed a comparatively lesser trend. SERr echoes the change observed in the ENS, indicating minimal change in identity structure of approximately ~0.1 SERr during the year which is likely consistent with year-round presence of certain species (figure 3B). Although each site exhibits seasonal variation of SERr, as the index approaches 0 it signifies less exchange meaning that there is a consistent acoustically ‘resident’ population of specific species or group of species.

The SERa ratio followed a consistent half-year turnover pattern at the five northernmost sites (Heezen to Wilmington canyons) but remains low, indicating minimal change in ‘dominance’ structure among cetacean species (figure 3C). The seasonal shift in dominance indicates that some species have a more significant acoustic presence during some months of the year and then are either less acoustically active or leave the area. This partial turnover indicates that some species are either migratory and leave the area or stop calling in a consistent seasonal pattern year to year, while other species remain year-round with no seasonal change in acoustic presence. To deduce the species driving these turnover patterns, we utilized additional methods that identify the acoustic presence of specific species.

### 3.3. Conditional inference trees

**Figure 4.**
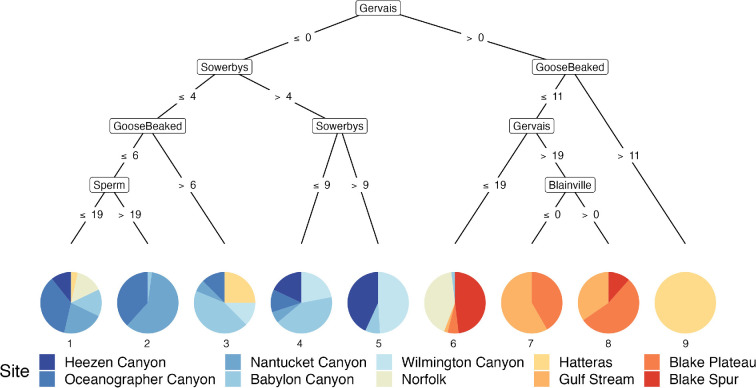
Conditional inference tree (Ctree) showing the partitioning effect of the presence of certain species (number of days per month) across 10 sites in the North Atlantic. Each Ctree node was restricted to a minimum sum of 60 weights and exceedance of a 0.95 test statistic. The size of the Ctree (depth) was not restricted, but the minimum sum of weights for each terminal node (numbered 1−9) was limited to 15. Each colour-coded site is labelled in the legend and ordered from north to south in order to latitude with a spectrum of colours from cool (blue) to warm (red). Beaked whale species represented all inner nodes except for one fourth tier node, which was sperm whales.

Conditional inference tree (Ctree) plots revealed the identity of specific species that are the most significant drivers of the seasonal patterns and changes in community groups across the 10 sites. When detections of all 13 analysed species and grouped delphinids were modelled in a full dataset Ctree, all the tree nodes were divided by odontocetes, and more specifically, all but one of the tree nodes were of beaked whale species (figure 4). The single non-beaked whale node was split by the presence or absence of sperm whales. Detections of Gervais' beaked whales (*M. europaeus*) were the primary distinguishing species (i.e. root node) and split sites according to presence or absence. Sites without detections of Gervais’ beaked whale were divided by general latitude in the mid-Atlantic with end nodes 1−5 being mostly comprised of sites that are north of the Maryland and Virginia border, while sites with detections of Gervais’ beaked whale were represented in nodes 6−9 and were mostly comprised of sites to the south (figure 4).

**Figure 5.**
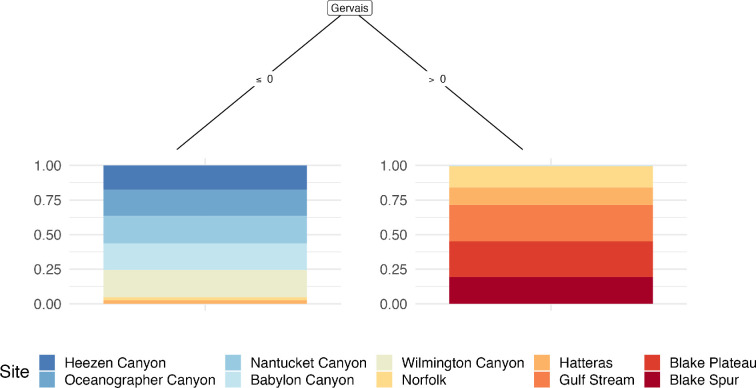
Conditional inference tree (Ctree) limited to a single split variable. By restricting the Ctree to a single node (stump control), the acoustic monitoring sites were divided into two terminal nodes. The bar graphs at each terminal node indicate the likelihood of the presence or absence of a single species, Gervais' beaked whales (*M. europaeus*), at each of the 10 sites in the North Atlantic. The stump Ctree split sites by latitude around 38°0′N, generally grouping the five sites to the north and five sites to the south. Babylon Canyon, Norfolk and Hatteras appeared in both terminal nodes, however, the likelihood of the presence (Norfolk and Hatteras) or absence (Babylon Canyon) is minimal compared to the contrary. Each colour-coded site is labelled in the legend and ordered from north to south in order of latitude with a spectrum of colours from cool (blue) to warm (red).

To visualize how Gervais' beaked whales split the 10 study sites, a second Ctree was generated with a stump (single node) restriction to limit the Ctree to only two end nodes (figure 5). By limiting the Ctree model it is apparent that Gervais' beaked whales divide the 10 study sites by latitude around 38°0’N, near the Norfolk and Hatteras sites, with no detections at sites north of Norfolk except for a single day (2016) across 3 years at Babylon Canyon. This result highlights the significance of the presence of Gervais' beaked whales to distinguish cetacean communities at these sites in the Atlantic Ocean.

In the full dataset Ctree, the nine pie chart end nodes show the likelihood of each site hosting the species names in the branches above (figure 4). In this Ctree, all but one of the end nodes (1–8) contains multiple sites which signifies that those sites are more similar to each other in terms of community composition. Only the 9th end node contained a single site, Hatteras, which followed the root node split on greater than 0 detections of Gervais' beaked whales to a node split on over 11 days per month of detections of goose-beaked whales. This result indicates that the presence of Gervais' and goose-beaked whales are uniquely significant to the cetacean acoustic community at Hatteras. We also observe that Norfolk and Hatteras appear in both ‘northern’ or blue-dominated and ‘southern’ or red-dominated end nodes, which reflects that the latitude of those sites is likely a transition zone between beaked whales species that are primarily detected at the more northern sites and beaked whales that are primarily detected at more southern sites. When Gervais’ beaked whales are not detected, Sowerby’s beaked whales were found to be important for differentiating Heezen Canyon, Oceanographer Canyon, Nantucket Canyon, Babylon Canyon, Wilmington Canyon from the five sites to the south (Norfolk Canyon, Hatteras, Gulf Stream, Blake Plateau and Blake Spur).

**Figure 6.**
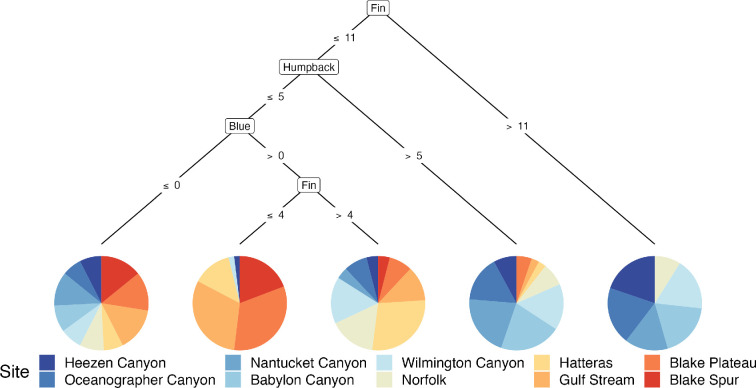
Conditional inference tree (Ctree) modelled from data in figure 4 to exclude all odontocete species. To compensate for the reduced data input, each Ctree node was restricted to a minimum sum of 10 weights and the minimum sum of weights for each terminal node was reduced to 5. As in figure 4, the size of the Ctree (depth) was not restricted and the exceedance of a test statistic was 0.95. Each colour-coded site is labelled in the legend and ordered from north to south in order of latitude with a spectrum of colours from cool (blue) to warm (red).

Due to the dominance of odontocetes in the Ctree built from the entire dataset, an additional model which excludes all odontocete species was generated to visualize any potential partitioning effect from mysticete species across the study sites (figure 6). In the mysticete-only Ctree the nodes are forced to divide out based only on detections of the baleen whale species included in analysis. However, in this model, the end nodes do not show a clear spatial pattern based on specific species as observed in the full dataset Ctree. Only fin, humpback and blue whales are included in the mysticete-only Ctree, as the model did not identify the presence of North Atlantic right whales nor sei whales to be significant in distinguishing individual sites. With only mysticete species, the Ctree does not show as strong a spatially related partitioning effect compared to when odontocetes are included (figure 4). This is likely due to differences in the spatial ecology and migratory behaviour of mysticete and odontocete species in the North Atlantic.

### 3.4. Acoustic niche

**Figure 7.**
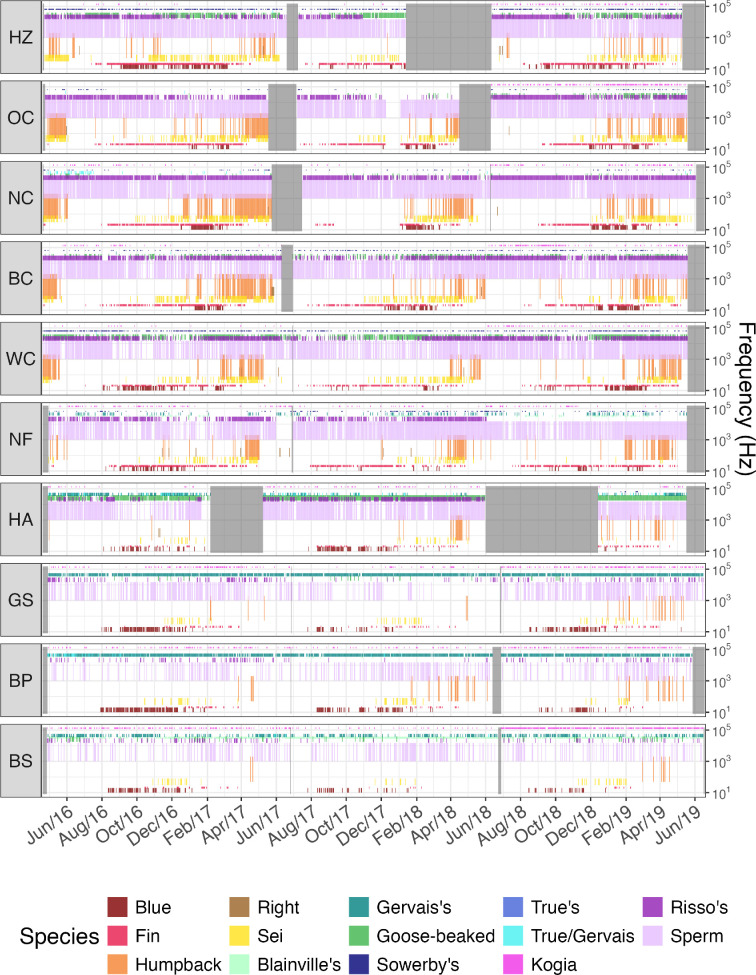
Spectrographic box display [38] for 10 passive acoustic monitoring sites in the North Atlantic from May 2016 to June 2019. Daily detections of 13 marine mammal species are shown by representative communication frequency ranges (for detailed table, see [39]). Mysticete species are labelled with red-orange colours, beaked whale species are shades of green and other odontocetes are labelled with blue-purple colours. Data gaps are represented with light grey shading (10 Hz–150 kHz).

Multi-year patterns and trends are summarized for each species and site in a spectrographic box display, depicting when and where specific species are acoustically detected (figure 7). The ‘acoustic niche’ of each species is also visualized as approximate call range for each species, differentiating species that are low-frequency specialists (e.g. blue whales) from species that are high-frequency specialists (e.g. Gervais' beaked whales). In separating the acoustic presence of individual species, the spectrographic box display illustrates the species that comprise cetacean communities at each site. For example, one or more beaked whale species were detected nearly every day at Hatteras and sites to the south, compared to a generally more sporadic acoustic presence at sites north of Hatteras.

At each of the sites, interannual patterns of species detections illuminate the community compositions driven by species with a year-round acoustic presence compared to species with more seasonal behaviour (e.g. presence of migratory species or species that are not consistently acoustically active throughout the year). Specifically, mysticete species (humpback, blue, fin, sei and right whales) were only detected during certain months of the year, which are slightly different for each species. In general, mysticetes were detected on more days at sites north of Hatteras. Risso’s dolphin and sperm whales were also detected on most days at the sites north of Hatteras, and comparatively less frequently at the three southernmost sites. In contrast, Kogia, the other odontocete species included in the analysis, were detected more frequently at the Blake Spur, Blake Plateau and Gulf Stream sites compared to the other more northern sites.

## 4. Discussion

In this article, we utilize PAM data to identify and compare community compositions of cetaceans in different soundscapes in the western North Atlantic. Using acoustic presence detections, we were able to combine new and established methods for PAM data analysis to visualize species diversity, temporal and spatial trends, and identify the species driving those differences. Combined with other efforts to assess cetacean presence and biodiversity, these complimentary community ecology assessments provide specific information about the individual environments and identify locations that appear to be important for different groupings of species. We build on previous work that evaluated seasonal presence of individual species at these same sites in the North Atlantic [17,40], to compare the significance of different groupings of present species at each site. We propose that future analyses can combine these metrics with other applications of PAM data, like soundscape monitoring and noise metrics, as well as other biodiversity monitoring efforts from visual surveys, eDNA, tagging and other methods to help us understand where animals are distributed over space and time, monitor long-term trends and identify drivers of diversity to aid in conservation.

### 4.1. Beaked whale species differentiate communities across sites

Specific beaked whale species varied meaningfully across the 10 sites. Reflecting previous work to document the spatial and temporal trends of beaked whale acoustic presence in the North Atlantic [17–19,40], our conditional inference tree model identified beaked whale species to be the most significant driver of differences among cetacean communities in the North Atlantic. This is likely because of the residency patterns and acoustic propagation range of these species, that is, different groupings of beaked whale species were detected across the sites, indicating that the latitudinal (spatial) range for each beaked whale species included in this analysis is narrower compared to other odontocete and mysticete species that were detected across all sites. Additionally, at some sites beaked whales were detected year-round. For example, Gervais’ beaked whales were detected year-round at sites south of Hatteras, illustrating the importance of those sites as habitat for the species. Gervais’ beaked whales were also identified to be the most significant driver of dissimilarity across sites in the conditional inference tree model (figures 4 and 5), emphasizing differences between sites to the north and south of 38°0′N around the Norfolk and Hatteras sites. Additionally, the presence of Gervais’ beaked whales has been linked to the presence of the Gulf Stream, which reflects how species monitoring can provide information about environmental conditions along the East Coast in addition to delineating cetacean communities [41].

Broadly, the conditional inference tree model demonstrates the importance of classifying beaked whales to species in cetacean survey and assessment efforts. We identified different species to be significant drivers of dissimilarity among community groups at a range of latitudes. In general, we observed that beaked whale species were less likely to be detected across a wide spatial range compared to baleen whales and other odontocete species. However, detections of baleen whale vocalizations were not uniformly distributed throughout all the sites in this comparison. Specifically, fewer baleen whale vocalizations were observed at the southernmost sites compared to sites at more northern latitudes. This difference is likely related to species specific migratory and calling behaviour.

To minimize detection bias among the species included in this analysis, detections were summarized in minimum time bins of 1 day. Although methods for acoustic detections of some species included more granular time scales, reporting a daily detection rate reduced the likelihood of missing (and therefore underreporting) calls at these shorter timescales. Similar to applications of duty-cycled sampling regimens, the probability of detection depends on species and call type [42–44]. While the trade-off of using a smaller time bin is a higher likelihood of underestimating animal presence, these results are still considered minimum estimates of presence across all species. Furthermore, call amplitude and environmental conditions are variable across space, time and species making it difficult, if not impossible, to control for variation in a comparison of this scale. However, the daily detection metric and use of a single recorder at each site minimizes such repetition.

### 4.2. Highly migratory species drive site dissimilarity over time

It was only possible to determine which mysticetes are more important in separating communities when all odontocete species were excluded from the conditional inference tree model (figure 6). However, none of the mysticetes species were significant drivers of differences among the cetacean communities assessed here. This aligns with what is already known about these highly migratory species moving through the different site locations [14]. It is also possible that under certain oceanographic conditions, calls from individual animals may have been detected at multiple sites. In the restricted conditional inference tree, fin whales were identified as more dominant than other mysticete species, perhaps because they vocalize in more repetitive song patterns over longer time periods compared to the acoustic behaviour of other species. Humpback and blue whales exhibit similar song behaviour during certain times of the year and were also represented as nodes. Neither sei nor North Atlantic right whales appear in the model, which may be due to a combination of fewer individuals of those species, calling from more coastal areas of the shelf-break and/or calling less frequently or at lower amplitudes compared to fin, blue and humpback whales.

Species exchange ratio analysis revealed that some of the species included in our analysis migrate to different habitats in a consistent seasonal pattern year to year, while other species remain within a smaller range without much seasonal change in acoustic activity. The SERa results show that the site-specific communities have annual cycles (figure 3C), which aligns with seasonal pattern observations in the spectrographic box display (figure 7). Trends of the SERa were slightly stronger (i.e. larger distance between highest and lowest values) compared to the SERr, indicating consistent partial turnover each half year at northern sites, with some species leaving the area in a consistent seasonal pattern year to year, while other species remain with minimal seasonal change (figure 3B). This is likely reflective of both the long-distance migratory and seasonal calling behaviour of the mysticete species, in contrast to the odontocete species that were detected via echolocation clicks that are considered to have a different function than mysticete song vocalizations. Due to the strong seasonality of mysticete vocalizations, these more transient species have a significant impact on the seasonality of soundscapes throughout the North Atlantic shelf break but are not meaningfully associated with any specific site or region (figure 6).

In contrast, sperm whales were consistently detected year-round at all sites. However, seasonal movement patterns and distribution of these animals are not as well documented as mysticete species—especially in the North Atlantic [45]. Thus, these results may indicate that certain groups are remaining in specific habitats throughout the year or could also suggest that the species is so ubiquitous within the North Atlantic that their distribution does not change across seasons in a meaningful way. While sperm whales were detected at all sites throughout the year, at the three southernmost sites detections were sparser with echolocation clicks being detected on comparably fewer days throughout the year. While sperm whales were not a significant driver of dissimilarity, their year-round acoustic presence contributes substantially to soundscapes throughout the North Atlantic shelf break (figure 7).

Seasonality of species’ acoustic behaviour may also imply acoustic niche partitioning [46] in specific time intervals or habitats. Niche theory proposes that species may avoid competition for acoustic communication space by limiting co-occurrence in overlapping frequency bands by either vocalizing in different frequencies or times (e.g. daily, seasonal). As observed in the spectrographic box display, peak acoustic daily detections of different mysticete species are lagged with no two species completely overlapping in seasonal occurrence (figure 7). In contrast, the observed spatial distribution of different beaked whale species may also be related to niche partitioning but with the distribution being spatial instead of temporal. This difference is a significant factor in the community composition of cetacean groups at the study sites. While the more nomadic species may drive differences within each site throughout the year (figure 3), it is the species with higher year-round site fidelity that define spatial dissimilarity of cetacean groups (figure 4).

### 4.3. Future applications

The analysis tools presented in this study are intended to demonstrate the opportunities of long-term passive acoustic monitoring (PAM) to monitor biodiversity over broad spatial scales. Although assessing community composition, species turnover and relative presence of species within defined environments is not novel, the methods to utilize PAM data and species-specific acoustic detections for more than a dozen cetacean species have not been clearly defined. Our goal was not to determine why or how different species may interact with other species and their habitats in different ways, but to utilize PAM data in a new approach to explore how different species interact with their acoustic environment. For example, exploring acoustic niche partitioning in the context of community assemblages may help explain species behaviour and co-occurrences that were previously poorly understood.

Although we only considered presence–absence data, future work could integrate other ecological applications as methods permit (e.g. density estimation) which may inform detailed evaluation of acoustic niches across groups of species as well as relationships between species richness and habitat covariates [47]. Additionally, community assemblage analyses may be valuable for assessing potentially different impacts of exposure to disturbance on species and can support decision making to minimize impacts of anthropogenic noise on sensitive species and environments. Consistent monitoring over time can be used to assess long-term patterns and trends of community assemblages, which may be especially valuable for tracking biodiversity in areas where potentially impactful anthropogenic activities overlap with data-deficient and/or protected species.

Monitoring acoustic communication space in tandem with other life-history needs (e.g. prey availability) can also provide information to deduce how different species may handle resource competition and if certain species exhibit resiliency that insinuates they are more adaptable to change (e.g. habitat or prey preferences). This information may be useful to managers and policy makers to prioritize conservation actions for certain species, communities, or habitats. Developing specialized tools and methods to efficiently filter and process raw data are critical to shorten the time needed between data sampling and reporting on the condition of an environment or status of a species. Needs for expedited reporting are likely to increase in coming years considering accelerating climate change, marine renewable energy development and endangered species monitoring.

## 5. Conclusion

The earth is a water planet, over 70% covered by the ocean, but of that vast expanse marine observing programs only manage to sample 7% of the ocean surface area [48]. The ocean is simply too big for current monitoring capacity. However, remote sensing tools such as PAM are a tremendous resource for observation over long time scales and widespread areas, and refining analysis tools can tap into existing datasets to reveal new information. Over the past few decades PAM tools have advanced significantly, making it easier and more economical for more scientists to utilize PAM data to answer a wide range of ecological research questions. Specific changes within ecosystems cannot be addressed until they are identified, and thus creative approaches are needed to expand monitoring and exploration without further demands on limited time and resources. Here, we demonstrate how a variety of community assemblage analysis methods can provide information about a broad swath of cetacean species and underwater monitoring areas to support marine resource management and conservation.

## Data Availability

Open Research Statement: Datasets utilized for this research are accessible via the NOAA Northeast Fisheries Science Center Passive Acoustic Cetacean Map v. 1.1.10: https://apps-nefsc.fisheries.noaa.gov/pacm and Dryad [49,50].
